# Clinical Outcomes for Emergency Department Presentations of Sepsis Managed on a Clinical Pathway: A Systematic Review and Meta-Analysis

**DOI:** 10.3390/healthcare14111509

**Published:** 2026-05-29

**Authors:** Andrew McKinlay, Giles Barrington, Sarah J. Prior, Viet Tran

**Affiliations:** 1 Royal Hobart Hospital, Tasmanian Health Service, Hobart 7000, Australia; giles.barrington@ths.tas.gov.au (G.B.); v.tran@utas.edu.au (V.T.); 2 TASER (Tasmanian Emergency Medicine Research) Institute, Hobart 7000, Australia; 3 Tasmanian School of Medicine, University of Tasmania, Hobart 7000, Australia; 4 Tasmanian School of Medicine, University of Tasmania, Burnie 7320, Australia; sarah.prior@utas.edu.au; 5 Menzies Institute for Medical Research, University of Tasmania, Hobart 7000, Australia

**Keywords:** systematic review, meta-analysis, sepsis, emergency, clinical pathway

## Abstract

**Background**: Sepsis is a time-critical condition requiring early recognition and intervention. Many emergency departments (EDs) have adopted clinical pathways to standardise sepsis care; however, the impact of these pathways on patient outcomes remains unclear. **Methods**: A systematic review and meta-analysis was conducted in November 2024 searching PubMed, Embase, and Scopus. Studies were included if they assessed the impact of a clinical pathway on adult or paediatric patients with sepsis presenting to the ED. **Results**: Thirty-three studies were included, of which the majority were retrospective cohort designs and were rated serious overall risk of bias. Pathway implementation was associated with faster time to antibiotics across all subgroups (135 min before vs. 93 min after; MD −43 min, *p* < 0.001). In-hospital mortality appeared reduced in the primary analysis (RD −2.4%, *p* = 0.032); however, this finding was fragile under sensitivity analysis and was not observed in prospective or randomised designs. The apparent reduction in hospital length of stay was driven by paediatric and low- and middle-income country studies and was non-significant when restricted to adult studies. ICU admission rate, ED length of stay, and time to IV fluid resuscitation were not significantly reduced. **Conclusions**: ED sepsis pathway implementation is associated with improved time to antibiotics across clinical settings and populations. Current evidence is insufficient to demonstrate a reduction in mortality; the apparent signal in retrospective studies is attributable to secular improvements in sepsis care and asymmetric patient identification rather than a true pathway effect. Future research should prioritise prospective controlled studies with standardised screening methods, time zero definitions and control of confounding variables.

## 1. Introduction

Sepsis is a syndrome of life-threatening organ dysfunction caused by a dysregulated host response to infection [1]. The sepsis mortality rate ranges from 15 to 50% [2], and in 2020 the WHO Global Report on Disease Burden and Mortality reported almost 50 million cases of sepsis with 11 million deaths [3]. Early recognition and treatment are the main principles of sepsis management. Observational studies have reported associations between antibiotic delay and increased mortality, with some studies finding a 7–9% increase in mortality per hour of delay [4,5].

A landmark paper by Rivers et al. in 2001 found a significant reduction in mortality following a bundle of interventions called early goal-directed therapy (EGDT), which involved invasive monitoring and aggressive treatment [6]. Subsequent multi-centre randomised controlled trials (RCTs) from 2014 to 2015 in the United States (ProCESS) [7], England (ProMISe) [8], and Australia (ARISE) [9] concluded that EGDT did not improve survival compared with less invasive protocols; however, it was clear that early recognition and the concept of bundled care were important to manage sepsis.

The Surviving Sepsis Campaign (SSC) is a global collaborative which represents over 25 expert international organisations and aims to reduce mortality and morbidity from sepsis [10]. The SSC’s most recent guideline published in 2021 recommends a group of interventions within one hour of patient presentation, including early sepsis identification, lactate measurement, blood cultures, antibiotics, and intravenous fluids [10]. Similarly, the 2022 Sepsis Clinical Care standard from the Australian Commission on Safety and Quality in Health Care (ACSQHC) recommends that patients with suspected sepsis are treated urgently according to a local clinical pathway [11]. The ACSQHC defines a clinical pathway as an intervention that supports clinician decision-making and care processes for a well-defined group of patients during a well-defined period. It suggests that pathways should include (a) criteria to support sepsis recognition, (b) triggers and time-frames for escalation of care, (c) guidance on appropriate interventions, and (d) time-frames to review response to treatment [11].

A key component of clinical pathway design is the screening tool used for suspected sepsis recognition. Although there is no single tool recommended by the SSC or the ACSQHC, scores often referenced in the literature include the quick sepsis-related organ failure assessment (qSOFA), systemic inflammatory response syndrome (SIRS) and national early warning score (NEWS). qSOFA has a high specificity of 82% but a low sensitivity of 46%, which means it performs well at predicting critical illness but has a high false-negative rate [12]. NEWS has an intermediate sensitivity of 73% and a specificity of 52%, and SIRS has the highest sensitivity of 82% but the lowest specificity at 24% [12].

Sepsis pathways are now commonly used in EDs across the world, but their impact on patient outcomes is poorly studied [13,14]. Rather than interrogating individual pathway components or compliance, this review focused on the impact of introducing a pathway compared with standard non-protocolised care.

We aimed to investigate whether the implementation of a standardised pathway results in improved process outcomes (time to antibiotics) and clinical outcomes (mortality, length of stay) in patients presenting to the ED with sepsis.

## 2. Materials and Methods

### 2.1. Search Strategy and Article Identification

Three databases (PubMed, Embase, Scopus) were searched in November 2024 using a three-component search term with variations and MeSH terms for “sepsis”, “emergency department” and “clinical pathway”. The review protocol was registered through Prospero (CRD42024618055), and the full search strategy is provided in Supplementary Table S1 [15]. Reference articles from the selected papers were screened by title and abstract and then by full-text review. Data were then extracted from papers included at this point. There were no restrictions imposed on publication date or country of origin, and only full-text papers available in English were included.

Included studies were related to adult and paediatric patients with sepsis presenting to the ED managed on a clinical pathway. A “clinical pathway” was defined as a paper or electronic tool which included: (a) screening criteria to identify patients with suspected sepsis, (b) triggers for escalation of care, (c) recommendations for sepsis interventions, and (d) time frames for review. Papers were excluded if they related to hospital-acquired infections. EGDT was also excluded as it was considered a specific treatment protocol for patients with severe sepsis rather than a local clinical pathway. Conference abstracts, trial registries and animal studies were not included.

### 2.2. Data Extraction and Quality Assessment

**Figure 1:**
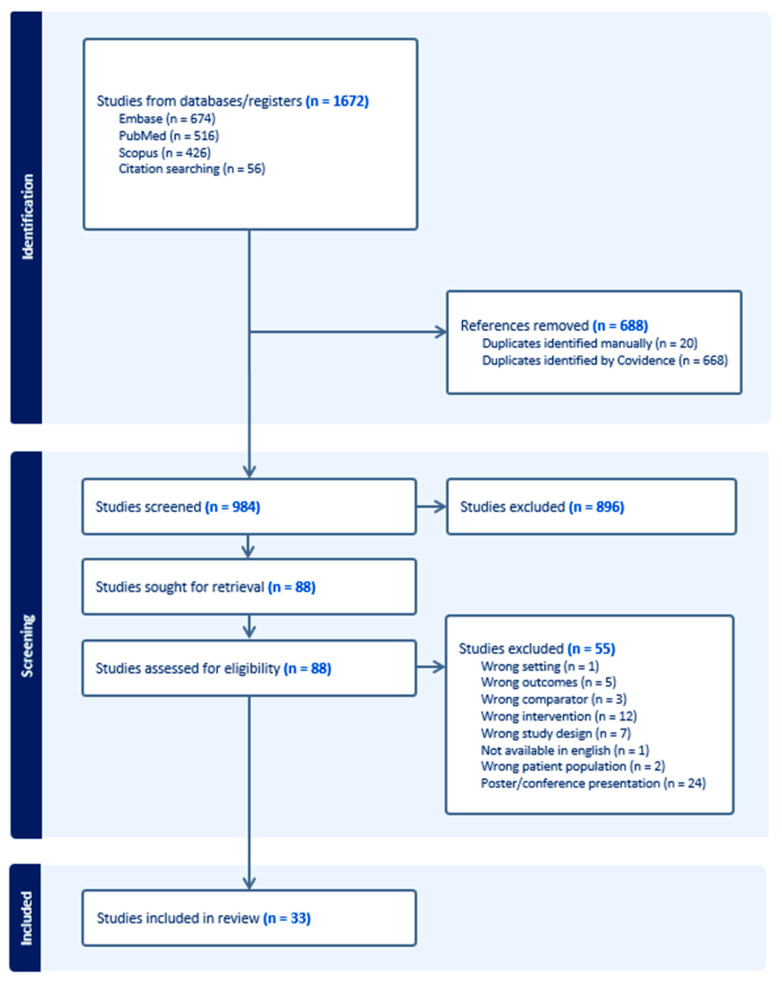
PRISMA flow diagram outlining the identification, screening and selection of final articles.

Titles and abstracts were independently screened by two reviewers to apply the criteria for study selection. Full text screening was performed by A.M. and G.B. with discrepancies resolved by consensus with V.T. Figure 1 displays the PRISMA flow diagram for final study inclusion and exclusion [16]. Data from included articles were extracted into a standardised Covidence extraction tool which included information related to study design, population demographics, pathway components, and patient outcomes [17]. For any missing data, an attempt was made to contact the corresponding authors.

Reporting completeness was assessed according to the Equator network’s Strengthening the Reporting of Observational Studies in Epidemiology (STROBE) Statement for observational studies [18], which is displayed in Supplementary Table S2. A risk of bias assessment was also undertaken using Cochrane’s “Risk of Bias in Non-randomised Studies of Interventions, Version 2” (ROBINS-I V2, Variant A, ITT) [19,20]. A.M. completed the initial risk assessment, which was verified independently by G.B. Although this review included one randomised trial, ROBINS-I rather than RoB-2 was utilised for consistency. Each study was graded by individual domain and overall risk of bias, which is reported in Supplementary Table S3. One study by Malhotra et al. [21] was found to have critical risk of bias and was excluded from all pooled analyses, consistent with ROBINS-I guidelines.

### 2.3. Statistical Analysis

Statistical analysis was conducted by S.P. in statistical software Stata18 (StataCorp LLC, Texas, USA), using metaprop and metan commands to pool estimates. For binary outcomes (mortality, ICU admission rate), data were pooled as risk differences (RD) using the DerSimonian–Laird (DL) random-effects estimator. For continuous outcomes (time to antibiotics, hospital LOS, ED LOS, and time to IV fluids), individual mean differences (MD) were pooled using the DL estimator. Statistical heterogeneity was assessed using the I^2^ statistic. Where studies reported medians with interquartile ranges, conversion was performed using the method of Wan et al. (2014) [22]. Studies reporting only a mean difference [23,24], or a median without interquartile range [25,26,27], could not contribute to the continuous pooled analyses. The 28-day and 30-day mortality were pooled as a combined endpoint given their comparable times and shared objective to measure post-discharge mortality at one month.

Sensitivity and subgroup analyses were conducted for the three primary outcomes (in-hospital mortality, time to antibiotics, hospital LOS) with stratification by (i) study design, (ii) overall risk of bias, (iii) patient population, and (iv) country income classification. Leave-one-out analyses were performed for each primary outcome to assess the influence of individual studies on pooled estimates.

## 3. Results

### 3.1. Study Characteristics

Thirty-three papers were analysed in this systematic review. The papers were primarily observational studies, including retrospective cohort studies (n = 19), prospective cohort studies (n = 6) and quasi-experimental studies (n = 5). There was one study that used pre–post simulation modelling [28], one interrupted time series [29] and one cluster randomised controlled trial [30].

Table 1 outlines the key article characteristics and sepsis pathway components, and an expanded version is included as Supplementary Table S4. The years of publication ranged from 2010 to 2024. The most common countries of origin were the United States of America (n = 11), Australia (n = 4) and Canada (n = 4). Nine studies were from countries classified as low- or middle-income. The ED population studied was primarily adults (n = 27), paediatric populations (n = 5), and one mixed ED [31]. Two-thirds of the papers were single-site interventions.

The design and delivery of each sepsis clinical pathway varied significantly. Twenty-five papers defined “time zero” (i.e., the time from which sepsis interventions must be completed) from patient arrival or triage. However, four papers instead considered time zero from when patients met sepsis screening criteria [28,30,34,53], one paper from the time of body fluid culture order [29], and another from the “time of recognition” [45]. Two papers did not state their screening tool or time zero [31,49].

The most common screening tool, used by thirteen papers, was a combination of “clinical concern for infection” and at least two SIRS criteria. Eleven papers used their own institution-specific criteria for altered vital signs and sepsis risk factors. Three papers used the qSOFA tool. Three papers only included patients if they had a subsequently confirmed infection, either by an adjudication committee [13] or positive blood cultures [47,51]. Eighteen papers used a paper-based screening tool, thirteen were electronic-based, and two did not state the mode of collection.

There was inconsistent reporting of possible confounding variables. Only 61% (n = 20) of studies described the initial severity of patient illness on arrival, 48% (n = 16) reported patient comorbidities, 64% (n = 21) the source of sepsis, and only 45% (n = 15) of studies performed subgroup analysis for patients with septic shock. Of those papers which did describe patient comorbidities, there were several different methods including the Elixhauser comorbidity index, Charlson comorbidity index and comorbidity point score version 2 (COPS2).

The ROBINS-I V2 tool was used to grade each paper’s overall risk of bias as low (3 papers, 9.1%), moderate (10 papers, 30.3%), serious (19 papers, 57.6%) or critical (1 paper, 3%). The most common domains in which bias was identified were Domain 1 (bias due to confounding) and Domain 3 (bias in selection of participants into the study). The complete assessment can be found in Supplementary Table S3.

The following clinical outcomes were reported by a corresponding number of papers: mortality (31, 94%), time to antibiotics (23, 70%), hospital length of stay (LOS) (21, 64%), rate of intensive care unit (ICU) admission (14, 42%) and ICU length of stay (10, 30%). Only nine publications (27%) reported on time to fluid resuscitation, six (18%) on volume of resuscitation and seven (21%) on rate of vasopressor use. Only six papers (18%) reported ED length of stay and five (15%) reported time to initial clinician assessment.

### 3.2. Study Design and Impact on Mortality

Before presenting pooled mortality estimates, it is important to highlight a consistent pattern which was identified when studies were grouped by design. Although the overall pooled in-hospital mortality estimate was statistically significant (RD −2.4%, *p* = 0.032), this finding was primarily driven by retrospective cohort studies. For the prospective cohort studies and the single randomised controlled trial, pathway implementation was not associated with reduced mortality at any endpoint. This pattern is likely explained by secular improvements in sepsis care over time, as well as asymmetric methods of patient identification between study periods. The mechanisms underlying this design-effect will be further discussed in Section 4.2.

### 3.3. Primary Outcomes

Full results are presented in Table 2 with subgroup analyses in Table 3. All binary outcomes are expressed as risk differences (RD) and continuous outcomes as mean differences (MD), with random-effects weighted means for pre- and post-intervention periods.

Mortality: The risk difference in 30-day/28-day mortality across eight studies was −2.9% (19.9% before vs. 17.0% after), which did not reach statistical significance. The pooled in-hospital mortality estimate across fifteen studies suggested a reduction of 2.4% (10.2% before vs. 7.8% after, *p* = 0.032), although this result was not replicated in prospective or randomised studies. The leave-one-out analysis revealed that this result was also susceptible to study exclusions, as omitting any one of five studies [29,33,39,47,49] caused the estimate to lose statistical significance. In the low/moderate risk of bias subgroup, the estimate was statistically significant but clinically small (RD −0.7%, *p* = 0.003), with both of the largest studies in this group showing null results. The estimate was also non-significant in adult studies (−1.5%, *p* = 0.086). In the context of the design-effect described above, the mortality findings in this review should be interpreted with caution.

Time to antibiotics: The most robust finding in this meta-analysis was a statistically significant reduction in time to antibiotics after sepsis pathway implementation. Across fifteen studies, pathway implementation was associated with a pooled reduction in time to antibiotics by 43 min (135 min before vs. 93 min after), which was significant in all subgroup analyses. Leave-one-out analysis confirmed all 15 iterations remained significant, with estimates ranging from −37 to −47 min.

Hospital length of stay: Although fifteen pooled studies showed an overall reduction in hospital length of stay from 6.8 days to 5.5 days (MD −1.3 days, *p* < 0.001), this result was not consistent across subgroups. The signal was primarily driven by paediatric and LMIC publications, and results became non-significant when restricted to adult-only (−0.2 days, *p* = 0.432) or low/moderate risk of bias studies (−0.5 days, *p* = 0.083).

ICU admission rate: Across twelve studies, the admission rate difference was −4.9% (23.5% before vs. 18.6% after), which did not reach statistical significance. Emergency department LOS and time to IV fluids were also not significantly reduced after sepsis pathway implementation.

## 4. Discussion

This systematic review aimed to examine the impact of sepsis pathway implementation on clinical and process outcomes compared with standard care. In this context, the intervention was defined as the presence or absence of the pathway itself, rather than compliance with or efficacy of individual pathway components.

Sepsis is a syndrome with no gold-standard diagnostic test [1], which was evident in the literature’s heterogeneous study populations, sepsis definitions, intervention components, and reporting of outcomes [54]. This review contained primarily retrospective cohort studies with poor control for confounders pre- and post-intervention. Factors such as patient comorbidities, illness severity and source of sepsis were not routinely evaluated, which contributed to over 60% of the included papers being graded as serious risk of bias. Overall, these factors make it challenging to draw strong conclusions about the outcomes of sepsis pathway implementation [13,14].

### 4.1. Improved Time to Antibiotics

The findings of this review support the conclusion that ED sepsis pathway implementation reduces time to antibiotic administration. The mean difference in antibiotic time pre- and post-intervention was 43 min (*p* < 0.001), which remained significant in every subgroup analysis (study design, risk of bias, patient population, and income setting). The substantial heterogeneity (I^2^ = 95%) likely reflects genuine variation in baseline antibiotic delivery times across settings, as well as methodological differences in pathway design, patient case mix, and screening tools used.

An important factor identified in this review was the variable definition of “time zero” from which sepsis interventions must be completed. Most studies considered triage time as “time zero”, but several instead used the time that a patient met sepsis criteria or the time of clinical recognition. Subgroup analysis by “time zero” definition could not be conducted due to insufficient studies for pooling in each group. This remains a significant methodological limitation in the literature, and future studies should standardise on a single “time zero” definition to improve comparability.

This variability in “time zero” definition may weaken the direct comparability of the pooled absolute times reported in Table 2. However, the primary pooled quantity is the within-study mean difference (the change in antibiotic administration time before and after pathway implementation), calculated using a consistent “time zero” applied to both periods within each individual study. The “time zero” convention therefore cancels within each study’s effect estimate, and the pooled estimate represents the improvement attributable to the intervention rather than a cross-study comparison of absolute times. The pooled absolute values should accordingly not be interpreted as directly comparable benchmarks across studies.

### 4.2. Mortality and Study Design-Effect

The most important finding to contextualise this review’s mortality results is the consistent difference between prospective and retrospective study designs. Although retrospective studies showed an apparent reduction in in-hospital mortality (RD −3.7%, *p* = 0.018), the prospective subgroup did not demonstrate any statistically significant reduction in either 30-day (RD −0.8%, *p* = 0.75) or in-hospital mortality (RD +0.6%, *p* = 0.62) after pathway implementation. The overall pooled in-hospital mortality estimate should therefore not be interpreted as evidence of a true pathway effect on mortality.

Two mechanisms are likely to account for this observed pattern. First, secular improvements in sepsis care over time are highly likely to confound all before–after studies without concurrent control groups. In retrospective studies, study periods were separated by several months to years, during which time developments in sepsis recognition and management occur. Without concurrent control groups, these background effects cannot be distinguished from pathway-specific effects. Only two studies in this review utilised contemporaneous control groups [28,32], and both found null mortality results. Secondly, the method of patient identification was inconsistent between groups. Pre-intervention patients were often captured by chart review using ICD-9 or ICD-10 codes for sepsis, whereas post-intervention patients were identified prospectively by the sepsis pathway itself. This method would be expected to capture more patients with lower severity illness in the post-intervention group, which may also result in an apparent reduction in mortality.

### 4.3. Hospital Length of Stay

Hospital length of stay was significantly reduced in the primary analysis (−1.3 days, *p* < 0.001) but again was not generalisable after subgroup analysis. The effect was not statistically significant when restricted to adult-only studies (−0.2 days, *p* = 0.432), prospective/RCT designs (+0.2 days, *p* = 0.399), or papers at low/moderate risk of bias (−0.5 days, *p* = 0.083).

The primary hospital LOS result was driven by two small studies which had disproportionate influence under random-effects weighting. A paediatric study showed a reduction of 8.7 days [52], and a LMIC study showed a reduction of 8.1 days [33]. LMIC settings have fundamentally different baseline lengths of stay, healthcare infrastructure and case mixes compared with adult EDs in high-income countries. This result is therefore not generalisable to overall pathway implementation and should be interpreted with caution.

### 4.4. Screening Tools and Patient Selection

Early detection of sepsis remains one of the most pertinent challenges in the field, and the current international sepsis guidelines only contain weak recommendations for sepsis screening [10]. Most studies in this review treated patients for sepsis if they met the criteria of “concern for infection” in combination with screening using SIRS (n = 13), an institution-specific tool (n = 11), or qSOFA (n = 3). This is another potential source of data heterogeneity as SIRS-based studies may capture a broader, lower-severity population than qSOFA-based studies, which would directly affect mortality rates and potentially mask pathway effects.

Some studies opted to only include patients with a “confirmed infection” such as via a positive blood culture. The concern with this methodology is that the rate of positive cultures is approximately 20–30% [23,47], and so clinically septic patients may have been excluded by this method. In contrast, some studies support that including a proportion of patients without confirmed infection indicates good external validity [30]. Overall, screened patients may or may not be ultimately diagnosed with sepsis or organ dysfunction, but they should be managed as if they have sepsis [30].

### 4.5. Emergency Department Outcomes

Despite sepsis pathways being predominantly an ED intervention, there were relatively few studies that reported emergency-specific outcomes. Neither ED LOS (MD −9.8 min, *p* = 0.249) nor time to IV fluids (MD −9.7 min, *p* = 0.206) were significantly reduced with pathway implementation, although both outcomes were limited to only six contributing studies. Length of stay is a complex metric which depends on factors such as bed availability, referral time, patient illness severity and time of day. It was also not possible to determine whether the volume of IV fluids received changed significantly after pathway implementation. This outcome was reported by several metrics including bolus volume, 3 h, 6 h or 24 h fluid totals. Only two studies reported fluid volumes in proportion to patient weight (mL/kg), which would also be useful for future standardisation.

Finally, there were mixed results when assessing whether sepsis pathways resulted in a more rapid patient review in ED. Two studies from Canada found there was a significant reduction in mean time to medical review from 74 min to 59 min (*p* = 0.01) [24] and from 100 min to 77 min (*p* < 0.001) [23]. Other papers found no impact on medical review time, perhaps because at baseline patients were being seen in under 30 min [42,43].

### 4.6. Intensive Care Outcomes

The pooled estimate for ICU admission rate did not reach statistical significance in the primary analysis (RD −4.9%, *p* = 0.1), although exploratory subgroup analysis found it was significant in low/moderate risk of bias studies (RD −4.1%, *p* = 0.005). This may support the principle that earlier recognition and antibiotic delivery can prevent progression to septic shock and intensive care admission. However, this post hoc analysis cannot establish causality from observational data, and the same design-effect mechanisms may apply to ICU outcomes.

Secular changes over the study periods may include changes to ICU admission thresholds, triage behaviour, improved identification of lower-acuity patients, and local policies regarding ward-level sepsis management. In general, there was also poor reporting of patient illness severity and the indications for ICU admission.

Other ICU outcomes that could not be pooled included rate of vasopressors and rate of intubation. Of the seven papers which reported vasopressor use, only one found a significant reduction in the post-intervention group [24], and two recent publications found no difference in the rate of mechanical ventilation [13,30]. The single large cluster-RCT in this review found no difference in any secondary ICU outcomes including acute heart failure within 24 h, number of days on mechanical ventilation, number of days on renal replacement therapy and vasopressor-free days [30].

### 4.7. Safety and Antimicrobial Stewardship

There is concern within the literature that protocols designed to accelerate sepsis treatment may result in excessive or unnecessary broad-spectrum antimicrobial use and increase subsequent adverse events [55]. This review identified only three papers which reported on adverse outcomes after protocol implementation. Seminari et al. found no difference in the rates of extended-spectrum beta-lactamase (ESBL) producers, Enterobacteriaceae carbapenemase-resistant infections or cases of *C. difficile* colitis [47]. Peltan et al. found that protocol implementation increased the chance of all ED patients receiving antibiotics (OR 1.08), but there was no increase in antimicrobial-related adverse events or *C. difficile* infections [32]. However, Flack et al. described a doubling of multidrug-resistant organism isolation rates (from 0.35% to 0.72%) following a QI initiative to increase early antibiotic use in the ED [50]. While the absolute rates were small and the study’s population was broad (all patients receiving broad-spectrum antibiotics), this finding supports the relationship between broad-spectrum antibiotic exposure and antimicrobial resistance. Further studies in this domain are needed and should consider the risks of antimicrobial resistance compared with the risks of delayed antibiotics.

### 4.8. Paediatrics

There were five studies that used paediatric sepsis pathways in this review. Time to antibiotics was significantly reduced in the paediatric subgroup (−39.4 min, *p* < 0.001), whereas there was no significant reduction in mortality (−5.0%, *p* = 0.431). There was high heterogeneity in this group, which may reflect the small sample size inherent to paediatric sepsis studies. The screening tools used to capture paediatric sepsis were less prescriptive and emphasised clinician or parental concern. The tools utilised a wide variety of risk factors including any altered vital signs, patient appearance, altered behaviour or level of consciousness, deterioration in current illness or patient re-presentation. None of the articles used a standardised clinical score.

Harley et al. reported ICU outcomes in children with sepsis and septic shock, and found that ICU LOS and mortality were comparable pre- and post-intervention, although there was a significant reduction in the need for interhospital transfer (41% vs. 12%, *p* = 0.01) [42]. The paediatric cohort had a lower acuity with fewer than 5% requiring ICU admission and a mortality of 1%. Almost half of the entire cohort did not receive any fluid bolus in the ED despite a presumptive diagnosis of sepsis, perhaps reflecting this low acuity, discomfort with intravenous access or prescribing of fluids in this population [42].

### 4.9. Low- and Middle-Income Countries

Although sepsis is a global problem, resource-limited countries have a higher burden of disease [56]. In this review, overall LMIC mortality ranged from 10% to as high as 24% in one pre-intervention cohort [44], with septic shock rates of up to 45% [31]. The reasons for this are multifactorial but may include delayed presentation of patients, lower socioeconomic conditions and pre-existing medical comorbidities [57].

This review included nine studies from low and middle-income countries, including India, Brazil, Tanzania, Pakistan, Haiti, Jordan and Thailand [21,25,31,33,34,37,44,45,49]. These publications were varied in their patient populations and reported outcomes and generally scored lower on quality assessment due to study size, data collection methods, non-equivalent groups and lack of consideration for study limitations.

The LMIC subgroup for antibiotic time (MD −27.7 min, *p* < 0.001, I^2^ = 0.0%) represents the most internally consistent finding in this review, with zero heterogeneity across four studies and a significant reduction from high baseline antibiotic times. Although the LMIC in-hospital mortality subgroup showed a significant reduction (RD −6.9%, *p* = 0.027), this was based predominantly on retrospective designs susceptible to the design-effect described in Section 4.2 and should be interpreted cautiously. Low and middle-income countries were also affected by pre-existing ED behaviours or supply issues such as a lack of available blood cultures or antibiotics in the department.

### 4.10. Limitations

This review has several limitations. First and most importantly, the majority of studies were retrospective pre–post designs without concurrent control groups, and therefore prone to selection bias, implementation bias, introduction of confounders, seasonality, and uneven clinical characteristics between groups. Global campaigns and improvements in sepsis care over time contribute to historical bias and inflate the perceived positive effect from sepsis pathway implementation. This limitation is the primary reason for the design-effect described previously and why results cannot be definitively attributed to pathways themselves.

Secondly, the methods of enrolment differed between groups, with most papers using ICD-9 or ICD-10 codes pre-intervention and sepsis screening tools post-intervention, creating an inherent selection bias. There may have been sepsis coding errors in the pre-intervention period, as well as susceptibility to overdiagnosis or false-positive screening in the post-intervention period. This was a major factor resulting in serious risk of bias in many studies, as it skewed data toward an apparent mortality benefit.

Thirdly, substantial heterogeneity across all outcomes (I^2^ = 65–95%) limits the interpretability of any single-pooled estimate. Sources of heterogeneity were largely uncontrollable, including variation in baseline care quality, pathway content, patient case mix, sepsis definition, screening tools and illness severity. Time zero definitions were also inconsistent between studies, which produced inherently different measurements. Although subgroup analyses were comparable across definitions, the pooled means should not be interpreted as a true reflection of sepsis care standards.

Formal GRADE assessment was not conducted, as the evidence in this review is considered of low certainty based on the predominance of non-randomised designs with high risk of bias and substantial heterogeneity across outcomes.

There were several other factors not directly addressed in this systematic review. The appropriateness of antibiotics was not analysed, as this is dependent on hospital location, sepsis source and local guidelines. We also did not address mortality as a function of compliance with the sepsis pathways, as it was the implementation of the pathway itself that was the primary intervention of concern. It is also beyond scope to review each individual pathway component, and it is acknowledged that there is still uncertainty around the evidence for and the timing of certain sepsis interventions [58].

## 5. Conclusions

ED sepsis pathway implementation is associated with faster antibiotic administration times across all study designs, patient populations and income settings. Prospective studies do not support an overall mortality benefit, and the apparent mortality signal in retrospective studies is most likely attributable to secular improvements and asymmetric case-selection rather than a true pathway effect. The reduction in hospital LOS in the primary analysis was driven by paediatric and LMIC studies and was not significant in adult or low risk of bias studies. These findings were based predominantly on studies with serious risk of bias and high heterogeneity, and should be interpreted with caution. Further high-quality prospective trials are needed to determine the true effectiveness of sepsis pathways, with a focus on standardised mortality endpoints, time zero definitions, measurement of confounding variables and comparison with concurrent control groups. Health services should continue to emphasise timely sepsis recognition, clinician judgement, and antimicrobial stewardship to optimise outcomes for patients presenting with sepsis to the ED.

## Figures and Tables

**Table 1:** Key article characteristics.

Study ID	Country	Design	Age	N	Sites	Time Zero	STROBE Score	Risk of Bias (ROBINS-I V2)	Reported Initial Illness Severity	Reported Comorbidities	Reported Sepsis Source	Subgroup Analysis for Septic Shock
Freund 2024 [30]	France; Spain	Cluster randomised controlled trial	Adult	872	23	Time of sepsis screening	22	Low	Yes	Yes	No	Yes
Lafon 2023 [13]	France	Quasi-experimental pre–post study	Adult	277	1	Triage	21	Serious	Yes	Yes	Yes	Yes
Peltan 2024 [32]	United States of America	Prospective cohort study	Adult	10151	3	Triage	22	Low	Yes	Yes	Yes	No
Medeiros 2021 [33]	Brazil	Retrospective cohort study	Paediatric	548	1	Triage	15	Serious	No	Yes	Yes	Yes
Taj 2022 [34]	Tanzania	Retrospective cohort study	Adult	126	1	Time of sepsis screening	15	Serious	No	Yes	Yes	No
Gatewood 2015 [35]	United States of America	Retrospective cohort study	Adult	761	1	Triage	15	Serious	No	No	No	No
Blythe 2022 [28]	Australia	Before and after simulation model	Paediatric	2030	48	Time of sepsis screening	19	Moderate	No	No	No	No
Pouryahya 2020 [36]	Australia	Prospective cohort study	Adult	191	3	Triage	13	Serious	No	No	No	No
Zia 2023 [37]	Pakistan	Retrospective cohort study	Adult	176	1	Triage	9	Serious	No	No	No	No
McColl 2017 [24]	Canada	Retrospective chart review	Adult	352	2	Triage	19	Moderate	Yes	Yes	No	No
Hayden 2016 [38]	United States of America	Retrospective cohort study	Adult	238	1	Triage	20	Serious	Yes	No	Yes	Yes
Threatt 2020 [39]	United States of America	Retrospective cohort study	Adult	310	1	Triage	11	Serious	No	No	No	No
Borguezam 2021 [31]	Brazil	Retrospective cohort study	Mixed	631	1	Not stated	15	Serious	Yes	No	Yes	Yes
McDonald 2018 [23]	Canada	Retrospective cohort study	Adult	634	1	Triage	19	Serious	No	No	Yes	No
Malhotra 2021 [21]	India	Retrospective cohort study	Adult	293	1	Triage	15	Critical	Yes	Yes	Yes	No
Noureldeen 2024 [40]	Saudi Arabia	Within-programme QI comparison	Adult	341	1	Triage	17	Serious	Yes	No	Yes	Yes
Liu 2016 [41]	United States of America	Retrospective cohort study	Adult	18,122	21	Triage	20	Moderate	Yes	Yes	No	Excluded
Barbash 2021 [29]	United States of America	Interrupted time series	Adult	54,225	11	Time that body fluid culture was ordered	22	Moderate	Yes	Yes	Yes	Yes
Harley 2021 [42]	Australia	Prospective cohort study	Paediatric	191	12	(1) Triage, and (2) SMO review	22	Serious	Yes	No	Yes	Yes
Moore 2019 [43]	United States of America	Retrospective cohort study	Adult	312	1	Triage	13	Serious	No	No	No	No
Papali 2017 [44]	Haiti	Retrospective cohort study	Adult	166	1	Triage	20	Serious	Yes	No	Yes	Yes
Bader 2020 [25]	Jordan	Quasi-experimental pre–post study	Adult	168	1	Triage	16	Serious	No	No	No	No
Mittal 2019 [45]	India	Quasi-experimental pre–post study	Paediatric	70	1	Time sepsis recognised by resident	16	Serious	No	Yes	Yes	No
Bruce 2015 [26]	United States of America; Canada	Retrospective cohort study	Adult	195	2	Triage	19	Moderate	Yes	No	Yes	No
Tromp 2010 [46]	The Netherlands	Prospective before–after study	Adult	825	1	Triage	18	Serious	No	No	Yes	Yes
Seminari 2023 [47]	Italy	Retrospective cohort study	Adult	722	1	Triage	16	Moderate	Yes	Yes	Yes	No
Song 2019 [48]	Korea	Quasi-experimental pre–post study	Adult	631	1	Triage	21	Low	Yes	Yes	Yes	Yes
Ruttanaseeha 2020 [49]	Thailand	Retrospective cohort study	Adult	192	1	Not stated	15	Serious	Yes	Yes	Yes	Yes
Francis 2010 [27]	Canada	Retrospective cohort study	Adult	213	3	Triage	20	Moderate	Yes	No	Yes	Yes
Flack 2023 [50]	United States of America	Retrospective cohort study	Adult	107	1	Triage	21	Serious	No	No	No	No
Venkatesh 2022 [51]	Australia	Prospective cohort study	Adult	1802	14	Triage	21	Moderate	Yes	Yes	Yes	Yes
Balamuth 2016 [52]	United States of America	Retrospective cohort study	Paediatric	189	1	Triage	21	Moderate	Yes	Yes	No	No
Narayanan 2016 [53]	United States of America	Prospective cohort study	Adult	214	1	Time of sepsis screening	18	Moderate	Yes	Yes	Yes	Yes

**Table 2:** Pooled estimates before vs. after sepsis pathway implementation. DerSimonian–Laird random-effects meta-analysis. N = number of studies. RD = risk difference (binary outcomes); MD = mean difference (continuous outcomes). Highlighted row (*) indicates *p* < 0.05.

Outcome	N	Before (Weighted Average)	After (Weighted Average)	Effect (95% CI)	*p*-Value	I^2^
30-day/28-day mortality	8	19.9%	17.0%	RD −2.9% (−6.3% to 0.4%)	0.086	85%
In-hospital mortality †	15	10.2%	7.8%	RD −2.4% (−4.7% to −0.2%)	0.032 *	65%
Time to antibiotics ‡	15	135 min	93 min	MD −43 min (−56 to −30)	<0.001 *	95%
Hospital LOS §	15	6.8 days	5.5 days	MD −1.3 days (−1.9 to −0.7)	<0.001 *	93%
ICU admission rate	12	23.5%	18.6%	RD −4.9% (−10.6% to 0.9%)	0.100	94%
ED LOS	6	271 min	261 min	MD −9.8 min (−26 to +7)	0.249	91%
Time to IV fluid	6	61 min	51 min	MD −9.7 min (−25 to +5)	0.206	89%

† Non-significant when restricted to prospective/RCT studies (RD 0.6%, *p* = 0.620). ‡ Barbash 2021 [29] excluded (time zero defined as culture-order). McColl 2017 [24] and McDonald 2018 [23] excluded (mean difference only reported). Bruce 2015 [26], Francis 2010 [27], and Bader 2020 [25] excluded (median only reported). Pooled absolute times should not be directly compared across studies with different time zero definitions. § Non-significant when restricted to adult studies (MD −0.18 days, *p* = 0.432) or low/moderate risk-of-bias studies (MD −0.5 days, *p* = 0.083).

**Table 3:** Subgroup analyses of primary outcomes. RD = risk difference (binary outcomes); MD = mean difference (continuous outcomes). Highlighted boxes indicate *p* < 0.05. N = number of studies. I^2^ indicates heterogeneity.

Subgroup	In-Hospital Mortality RD (95% CI), p, N, I^2^	Time to Antibiotics MD (95% CI), p, N, I^2^	Hospital LOS MD (95% CI), p, N, I^2^
All eligible studies	−2.4% (−4.7%, −0.2%), N = 15	−43 min (−56, −30), N = 15	−1.3 days (−1.9, −0.7), N = 15
Study Design			
Prospective/RCT	+0.6% (−1.8%, 3.1%), N = 3	−59 min (−93, −25), N = 5	+0.2 days (−0.2, 0.6), N = 5
Retrospective cohort	−3.7% (−6.7%, −0.6%), N = 11	−42 min (−63, −21), N = 8	−2.5 days (−4.0, −1.0), N = 6
Quasi-experimental	N = 1 (insufficient for pooling)	−15 min (−35, 5.7), N = 2	−2.7 days (−5.0, −0.4), N = 4
Risk of bias			
Low/Moderate	−0.7% (−1.2%, −0.2%), N = 6	−36 min (−52, −20), N = 7	−0.5 days (−1.1, 0.1), N = 7
Serious	−3.2% (−7.9%, 1.5%), N = 9	−51 min (−72, −29), N = 8	−2.3 days (−4.7, 0.01), N = 8
Population			
Adult	−1.5% (−3.2%, 0.2%), N = 12	−44 min (−59, −29), N = 11	−0.2 days (−0.6, 0.3), N = 10
Paediatric	−5.0% (−17.5%, 7.5%), N = 3	−39 min (−60, −18), N = 4	−4.6 days (−8.9, −0.4), N = 5
Income setting			
High-income countries	−0.8% (−2.3%, 0.6%), N = 10	−50 min (−66, −34), N = 11	−0.6 days (−1.1, −0.1), N = 12
Low/middle-income	−6.9% (−13.1%, −0.8%), N = 5	−28 min (−35, −21), N = 4	−4.6 days (−10.2, 1.0), N = 3

## Data Availability

No new data were created or analysed in this study. Data sharing is not applicable to this article.

## Supplementary Materials

The following supporting information can be downloaded at: https://www.mdpi.com/article/10.3390/healthcare14111509/s1, Supplementary Table S1: Search Strategy; Supplementary Table S2: STROBE Scores; Supplementary Table S3: ROBINS Risk of Bias; Supplementary Table S4: Expanded Study Characteristics.

## Abbreviations

The following abbreviations are used in this manuscript:

ACSQHC	Australian Commission on Safety and Quality in Health Care
CI	Confidence Interval
COPS2	Comorbidity Point Score, Version 2
ED	Emergency Department
EGDT	Early Goal-Directed Therapy
ESBL	Extended-Spectrum Beta-Lactamase
I^2^	Statistical measure of heterogeneity
ICD-9	International Classification of Diseases, 9th Revision
ICD-10	International Classification of Diseases, 10th Revision
ICU	Intensive Care Unit
IQR	Interquartile Range
IV	Intravenous
LMIC	Low- and Middle-Income Country
LOS	Length of Stay
MD	Mean Difference
MDR	Multidrug-Resistant
MeSH	Medical Subject Headings
NEWS	National Early Warning Score
OR	Odds Ratio
PRISMA	Preferred Reporting Items for Systematic Reviews and Meta-Analyses
PROSPERO	International Prospective Register of Systematic Reviews
QI	Quality Improvement
qSOFA	Quick Sepsis-Related Organ Failure Assessment
RD	Risk Difference
ROBINS-I	Risk of Bias in Non-Randomised Studies of Interventions
SIRS	Systemic Inflammatory Response Syndrome
SSC	Surviving Sepsis Campaign
STROBE	Strengthening the Reporting of Observational Studies in Epidemiology
WHO	World Health Organization

## References

[1] Singer M., Deutschman C.S., Seymour C.W., Shankar-Hari M., Annane D., Bauer M., Bellomo R., Bernard G.R., Chiche J.-D., Coopersmith C.M. (2016). The Third International Consensus Definitions for Sepsis and Septic Shock (Sepsis-3). JAMA.

[2] Bauer M., Gerlach H., Vogelmann T., Preissing F., Stiefel J., Adam D. (2020). Mortality in sepsis and septic shock in Europe, North America and Australia between 2009 and 2019—Results from a systematic review and meta-analysis. Crit. Care.

[3] Rudd K.E., Johnson S.C., Agesa K.M., Shackelford K.A., Tsoi D., Kievlan D.R., Colombara D.V., Ikuta K.S., Kissoon N., Finfer S. (2020). Global, regional, and national sepsis incidence and mortality, 1990–2017: Analysis for the Global Burden of Disease Study. Lancet.

[4] Ferrer R., Martin-Loeches I., Phillips G., Osborn T.M., Townsend S., Dellinger R.P., Artigas A., Schorr C., Levy M.M. (2014). Empiric antibiotic treatment reduces mortality in severe sepsis and septic shock from the first hour: Results from a guideline-based performance improvement program. Crit. Care Med..

[5] Liu V.X., Fielding-Singh V., Greene J.D., Baker J.M., Iwashyna T.J., Bhattacharya J., Escobar G.J. (2017). The Timing of Early Antibiotics and Hospital Mortality in Sepsis. Am. J. Respir. Crit. Care Med..

[6] Rivers E., Nguyen B., Havstad S., Ressler J., Muzzin A., Knoblich B., Peterson E., Tomlanovich M. (2001). Early goal-directed therapy in the treatment of severe sepsis and septic shock. N. Engl. J. Med..

[7] Yealy D.M., Kellum J.A., Huang D.T., Barnato A.E., Weissfeld L.A., Pike F., Terndrup T., Wang H.E., Hou P.C., LoVecchio F. (2014). A randomized trial of protocol-based care for early septic shock. N. Engl. J. Med..

[8] Mouncey P.R., Osborn T.M., Power G.S., Harrison D.A., Sadique M.Z., Grieve R.D., Jahan R., Harvey S.E., Bell D., Bion J.F. (2015). Trial of early, goal-directed resuscitation for septic shock. N. Engl. J. Med..

[9] Peake S.L., Delaney A., Bailey M., Bellomo R., Cameron P.A., Cooper D.J., Higgins A.M., Holdgate A., Howe B.D., Webb S.A. (2014). Goal-directed resuscitation for patients with early septic shock. N. Engl. J. Med..

[10] Evans L., Rhodes A., Alhazzani W., Antonelli M., Coopersmith C.M., French C., Machado F.R., McIntyre L., Ostermann M., Prescott H.C. (2021). Surviving sepsis campaign: International guidelines for management of sepsis and septic shock 2021. Intensive Care Med..

[11] Australian Commission on Safety and Quality in Health Care (2022). Sepsis Clinical Care Standard.

[12] Wang C., Xu R., Zeng Y., Zhao Y., Hu X. (2022). A comparison of qSOFA, SIRS and NEWS in predicting the accuracy of mortality in patients with suspected sepsis: A meta-analysis. PLoS ONE.

[13] Lafon T., Baisse A., Karam H.H., Organista A., Boury M., Otranto M., Blanchet A., Daix T., François B., Vignon P. (2023). Sepsis Unit in the Emergency Department: Impact on Management and Outcome of Septic Patients. Shock.

[14] Uffen J.W., Oosterheert J.J., Schweitzer V.A., Thursky K., Kaasjager H.A.H., Ekkelenkamp M.B. (2021). Interventions for rapid recognition and treatment of sepsis in the emergency department: A narrative review. Clin. Microbiol. Infect..

[15] PROSPERO (2024). Clinical Outcomes for Emergency Department Presentations of Sepsis Managed on a Clinical Pathway: A Systematic Review and Meta-Analysis. https://www.crd.york.ac.uk/PROSPERO/view/CRD42024618055.

[16] Page M.J., McKenzie J.E., Bossuyt P.M., Boutron I., Hoffmann T.C., Mulrow C.D., Shamseer L., Tetzlaff J.M., Akl E.A., Brennan S.E. (2021). The PRISMA 2020 statement: An updated guideline for reporting systematic reviews. BMJ.

[17] Veritas Health Innovation (2024). Covidence Systematic Review Software.

[18] von Elm E., Altman D.G., Egger M., Pocock S.J., Gøtzsche P.C., Vandenbroucke J.P. (2007). Strengthening the Reporting of Observational Studies in Epidemiology (STROBE) statement: Guidelines for reporting observational studies. BMJ.

[19] Sterne J.A., Hernán M.A., Reeves B.C., Savović J., Berkman N.D., Viswanathan M., Henry D., Altman D.G., Ansari M.T., Boutron I. (2016). ROBINS-I: A tool for assessing risk of bias in non-randomised studies of interventions. BMJ.

[20] Sterne J., Higgins J. (2025). ROBINS-I V2 Tool. https://www.riskofbias.info/welcome/robins-i-v2.

[21] Malhotra C., Kumar A., Sahu A.K., Ramaswami A., Bhoi S., Aggarwal P., Lodha R., Kapil A., Vaid S., Joshi N. (2021). Strengthening sepsis care at a tertiary care teaching hospital in New Delhi, India. BMJ Open Qual..

[22] Wan X., Wang W., Liu J., Tong T. (2014). Estimating the sample mean and standard deviation from the sample size, median, range and/or interquartile range. BMC Med. Res. Methodol..

[23] McDonald C.M., West S., Dushenski D., Lapinsky S.E., Soong C., van den Broek K., Ashby M., Wilde-Friel G., Kan C., McIntyre M. (2018). Sepsis now a priority: A quality improvement initiative for early sepsis recognition and care. Int. J. Qual. Health Care.

[24] McColl T., Gatien M., Calder L., Yadav K., Tam R., Ong M., Taljaard M., Stiell I. (2017). Implementation of an Emergency Department Sepsis Bundle and System Redesign: A Process Improvement Initiative. Can. J. Emerg. Med..

[25] Bader M.Z., Obaid A.T., Al-Khateb H.M., Eldos Y.T., Elaya M.M. (2020). Developing Adult Sepsis Protocol to Reduce the Time to Initial Antibiotic Dose and Improve Outcomes among Patients with Cancer in Emergency Department. Asia-Pac. J. Oncol. Nurs..

[26] Bruce H.R., Maiden J., Fedullo P.F., Kim S.C. (2015). Impact of nurse-initiated ED sepsis protocol on compliance with sepsis bundles, time to initial antibiotic administration, and in-hospital mortality. J. Emerg. Nurs..

[27] Francis M., Rich T., Williamson T., Peterson D. (2010). EM Advances: Effect of an emergency department sepsis protocol on time to antibiotics in severe sepsis. Can. J. Emerg. Med..

[28] Blythe R., Lister P., Seaton R., Harley A., Schlapbach L.J., McPhail S., Venkatesh B., Irwin A., Raman S. (2022). Patient and economic impact of implementing a paediatric sepsis pathway in emergency departments in Queensland, Australia. Sci. Rep..

[29] Barbash I.J., Davis B.S., Yabes J.G., Seymour C.W., Angus D.C., Kahn J.M. (2021). Treatment Patterns and Clinical Outcomes After the Introduction of the Medicare Sepsis Performance Measure (SEP-1). Ann. Intern. Med..

[30] Freund Y., Cancella de Abreu M., Lebal S., Rousseau A., Lafon T., Yordanov Y., Macrez R., Coisy F., Le Borgne P., Femy F. (2024). Effect of the 1-h bundle on mortality in patients with suspected sepsis in the emergency department: A stepped wedge cluster randomized clinical trial. Intensive Care Med..

[31] Borguezam C.B., Sanches C.T., Albaneser S.P.R., Moraes U.R.O., Grion C.M.C., Kerbauy G. (2021). Managed clinical protocol: Impact of implementation on sepsis treatment quality indicators. Rev. Bras. Enferm..

[32] Peltan I.D., Bledsoe J.R., Jacobs J.R., Groat D., Klippel C., Adamson M., Hooper G.A., Tinker N.J., Foster R.A., Stenehjem E.A. (2024). Effectiveness and Safety of an Emergency Department Code Sepsis Protocol: A Pragmatic Clinical Trial. Ann. Am. Thorac. Soc..

[33] Medeiros D.N.M., Mafra A., Carcillo J.A., Troster E.J. (2021). A Pediatric Sepsis Protocol Reduced Mortality and Dysfunctions in a Brazilian Public Hospital. Front. Pediatr..

[34] Taj M., Kassamali S.A., Khan Jiwani B., Sulaiman Khan Z., Pandian V. (2022). Outcomes of evidence-based modified sepsis protocol in an emergency department in Tanzania. Int. Emerg. Nurs..

[35] Gatewood M.O., Wemple M., Greco S., Kritek P.A., Durvasula R. (2015). A quality improvement project to improve early sepsis care in the emergency department. BMJ Qual. Saf..

[36] Pouryahya P., Guiney N., Meyer A., Goldie N. (2020). Evaluating the implementation and outcomes of a sepsis pathway in the emergency department. N. Z. Med. J..

[37] Zia I., Zaidi S.K.F. (2023). Promoting Antibiotic Stewardship and Implementation of Sepsis Pathway in the Emergency Department: A Quality Improvement Initiative. Cureus.

[38] Hayden G.E., Tuuri R.E., Scott R., Losek J.D., Blackshaw A.M., Schoenling A.J., Nietert P.J., Hall G.A. (2016). Triage sepsis alert and sepsis protocol lower times to fluids and antibiotics in the ED. Am. J. Emerg. Med..

[39] Threatt D.L. (2020). Improving Sepsis Bundle Implementation Times: A Nursing Process Improvement Approach. J. Nurs. Care Qual..

[40] Noureldeen H., Bakhsh A., Alshabasy A., Alawi M., Bakhribah A., Nasrallah N., Aljuhani O., Margushi R., Bantan R., Bokhari R. (2024). Enhancing Sepsis Care at an Academic Emergency Department in a Resource-Constrained Setting: A Quality Improvement Initiative. J. Patient Saf..

[41] Liu V.X., Morehouse J.W., Marelich G.P., Soule J., Russell T., Skeath M., Adams C., Escobar G.J., Whippy A. (2016). Multicenter implementation of a treatment bundle for patients with sepsis and intermediate lactate values. Am. J. Respir. Crit. Care Med..

[42] Harley A., Lister P., Gilholm P., Rice M., Venkatesh B., Johnston A.N.B., Massey D., Irwin A., Gibbons K., Schlapbach L.J. (2021). Queensland Pediatric Sepsis Breakthrough Collaborative: Multicenter Observational Study to Evaluate the Implementation of a Pediatric Sepsis Pathway Within the Emergency Department. Crit. Care Explor..

[43] Moore W.R., Vermuelen A., Taylor R., Kihara D., Wahome E. (2019). Improving 3-Hour Sepsis Bundled Care Outcomes: Implementation of a Nurse-Driven Sepsis Protocol in the Emergency Department. J. Emerg. Nurs..

[44] Papali A., Eoin West T., Verceles A.C., Augustin M.E., Nathalie Colas L., Jean-Francois C.H., Patel D.M., Todd N.W., McCurdy M.T. (2017). Treatment outcomes after implementation of an adapted WHO protocol for severe sepsis and septic shock in Haiti. J. Crit. Care.

[45] Mittal Y., Sankar J., Dhochak N., Gupta S., Lodha R., Kabra S.K. (2019). Decreasing the Time to Administration of First Dose of Antibiotics in Children With Severe Sepsis. J. Healthc. Qual..

[46] Tromp M., Hulscher M., Bleeker-Rovers C.P., Peters L., van den Berg D.T., Borm G.F., Kullberg B.J., van Achterberg T., Pickkers P. (2010). The role of nurses in the recognition and treatment of patients with sepsis in the emergency department: A prospective before-and-after intervention study. Int. J. Nurs. Stud..

[47] Seminari E., Colaneri M., Corbella M., De Silvestri A., Muzzi A., Perlini S., Martino I.F., Marvulli L.N., Arcuri A., Maffezzoni M. (2023). Reduction of BSI associated mortality after a sepsis project implementation in the ER of a tertiary referral hospital. Sci. Rep..

[48] Song J., Cho H., Park D.W., Ahn S., Kim J.Y., Seok H., Park J., Moon S. (2019). The effect of the intelligent sepsis management system on outcomes among patients with sepsis and septic shock diagnosed according to the Sepsis-3 definition in the emergency department. J. Clin. Med..

[49] Ruttanaseeha W., Hurnmek S., Ienghong K., Gaysonsiri D., Apiratwarakul K., Bhudhisawasdi V. (2020). Implementation of sepsis protocol for timely antibiotic administration in the emergency department. J. Med. Assoc. Thai..

[50] Flack T., Oaxaca D.M., Olson C.M., Pafford C., Strachan C.C., Epperson D.W., Reyes J., Akinrotimi D., Ho L., Hunter B.R. (2023). Association of a sepsis initiative on broad spectrum antibiotic use and outcomes in an ED population: A retrospective cohort study. Am. J. Emerg. Med..

[51] Venkatesh B., Schlapbach L., Mason D., Wilks K., Seaton R., Lister P., Irwin A., Lane P., Redpath L., Gibbons K. (2022). Impact of 1-hour and 3-hour sepsis time bundles on patient outcomes and antimicrobial use: A before and after cohort study. Lancet Reg. Health West. Pac..

[52] Balamuth F., Weiss S.L., Fitzgerald J.C., Hayes K., Centkowski S., Chilutti M., Grundmeier R.W., Lavelle J., Alpern E.R. (2016). Protocolized treatment is associated with decreased organ dysfunction in pediatric severe sepsis. Pediatr. Crit. Care Med..

[53] Narayanan N., Gross A.K., Pintens M., Fee C., MacDougall C. (2016). Effect of an electronic medical record alert for severe sepsis among ED patients. Am. J. Emerg. Med..

[54] Kramer R.D., Cooke C.R., Liu V., Miller R.R., Iwashyna T.J. (2015). Variation in the Contents of Sepsis Bundles and Quality Measures. A Systematic Review. Ann. Am. Thorac. Soc..

[55] Klompas M., Calandra T., Singer M. (2018). Antibiotics for Sepsis—Finding the Equilibrium. JAMA.

[56] Papali A., McCurdy M.T., Calvello E.J. (2015). A “three delays” model for severe sepsis in resource-limited countries. J. Crit. Care.

[57] Jacob S.T., Lim M., Banura P., Bhagwanjee S., Bion J., Cheng A.C., Cohen H., Farrar J., Gove S., Hopewell P. (2013). Integrating sepsis management recommendations into clinical care guidelines for district hospitals in resource-limited settings: The necessity to augment new guidelines with future research. BMC Med..

[58] Seymour C.W., Gesten F., Prescott H.C., Friedrich M.E., Iwashyna T.J., Phillips G.S., Lemeshow S., Osborn T., Terry K.M., Levy M.M. (2017). Time to Treatment and Mortality during Mandated Emergency Care for Sepsis. N. Engl. J. Med..

