# Supplementary material for: Clinical Outcomes for Emergency Department Presentations of Sepsis Managed on a Clinical Pathway: A Systematic Review and Meta-Analysis
Source: Healthcare (Basel). 2026 May 29;14(11):1509. doi: 10.3390/healthcare14111509 (PMC13256114; doi:10.3390/healthcare14111509)
Supplement: Supplementary file 1 [file healthcare-14-01509-s001.zip › Supplemental Table S1. Search Strategy.pdf]

Supplemental Table S1: Literature Review Search Terms and Results According to Database

Database	Search Terms	Results
PubMed	(((Sepsis[Mesh]) OR (Sepsis OR "septic shock" OR SIRS OR "systemic inflammatory response syndrome" OR "bloodstream infection" OR septicaemia OR bacteraemia)) AND (("Emergency Medicine"[Mesh] OR "Emergency Service, Hospital"[Mesh]) OR ("emergency medicine" OR "emergency department" OR "Trauma Cent*" OR "Emergency Unit" OR "Emergency Room"))) AND (("Critical Pathways"[Mesh]) OR ("sepsis pathway" OR package OR bundle* OR "sepsis protocol"))	516
Embase	'exp sepsis/':ti,ab,kw OR sepsis:ti,ab,kw OR 'septic shock':ti,ab,kw OR sirs:ti,ab,kw OR 'systemic inflammatory response syndrome':ti,ab,kw OR 'bloodstream infection':ti,ab,kw OR septicaemia:ti,ab,kw OR bacteraemia:ti,ab,kw<br>AND<br>'exp emergency medicine/':ti,ab,kw OR 'exp emergency ward/':ti,ab,kw OR 'emergency medicine':ti,ab,kw OR 'emergency department':ti,ab,kw OR 'trauma cent*':ti,ab,kw OR 'emergency unit':ti,ab,kw OR 'emergency room':ti,ab,kw<br>AND<br>'exp clinical pathway/':ti,ab,kw OR 'sepsis pathway':ti,ab,kw OR package:ti,ab,kw OR bundle*:ti,ab,kw OR 'sepsis protocol':ti,ab,kw	674
Scopus	(TITLE-ABS-KEY (sepsis OR "septic shock" OR sirs OR "systemic inflammatory response syndrome" OR "bloodstream infection" OR septicaemia OR bacteraemia)) AND (TITLE-ABS-KEY ("emergency medicine" OR "emergency department" OR "Trauma Cent*" OR "Emergency Unit" OR "Emergency Room")) AND (TITLE-ABS-KEY ("sepsis pathway" OR package OR bundle* OR "sepsis protocol"))	426
