# Supplementary material for: Clinical Outcomes for Emergency Department Presentations of Sepsis Managed on a Clinical Pathway: A Systematic Review and Meta-Analysis
Source: Healthcare (Basel). 2026 May 29;14(11):1509. doi: 10.3390/healthcare14111509 (PMC13256114; doi:10.3390/healthcare14111509)
Supplement: Supplementary file 1 [file healthcare-14-01509-s001.zip › Supplemental Table S3. ROBINS Risk of Bias.pdf]

ROBINS-I V2 Assessment Grid

Clinical outcomes for Emergency Department presentations of sepsis managed on a clinical pathway | PROSPERO: CRD42024618055 | Authors: McKinlay, Barrington, Prior, Tran

Tool: ROBINS-I V2 Variant A (ITT) applied to all 33 studies | Primary outcome: All-cause in-hospital mortality (LOS/process outcomes where mortality not reported)

#	Author (Year)	Study Design & Setting	D1 Confound	D2 Classif	D3 Selection	D4 Missing Data	D5 Outcomes	D6 Reporting	Overall	Outcome and Notes
1	Freund 2024	Cluster RCT (stepped-wedge) France/Spain N=872	Low	Low	Low	Low	Low	Low	Low	Outcome: Mortality (aRR 0.81 [0.48–1.39], p=NR) All domains Low. Stepped-wedge RCT with GLMM + period/cluster adjustment. Primary limitation: underpowered (~75% of target N=1,148); null result cannot exclude clinically meaningful benefit.
2	Lafon 2023	Quasi-experimental pre-post France N=277	Serious	Moderate	Serious	Low	Low	Moderate	Serious	Outcome: Day-28 mortality (40% vs 28%, p=0.013) **D1 Serious:** No adjustment despite SOFA H0 5.8 vs 4.7 (p<0.001) and MAP 72 vs 82 mmHg (p<0.001). **D2 Moderate:** ICD vs physician-activation asymmetry. **D3 Serious:** Adjudication committee confirmation + blood culture rate increase → differential cohort selection between phases. **D6 Moderate:** No trial registration; protocol differences between phases not specified in advance.
3	Peltan 2024	Pragmatic trial (DiD, 3 EDs) USA N=10,151	Low	Low	Low	Low	Low	Low	Low	Outcome: 30-day mortality (aOR 0.90 [0.68–1.19], p=0.45) All domains Low. Most rigorous study in review: DiD + contemporaneous controls, a priori DAG confounders, parallel trends formally tested, ITS sensitivity, death registry ascertainment, prospective registration.
4	Medeiros 2021	Retrospective cohort (paediatric LMIC) Brazil N=548	Serious	Serious	Serious	Low	Low	Moderate	Serious	Outcome: In-hospital mortality (RR 0.13 [0.06–0.27], p<0.001, propensity-adjusted) **D1 Serious:** Omitted septic shock (45.8% vs 1.6%); clinically significant infant vs toddler difference (8.75 vs 19 months, p<0.001, undiscussed). **D2 Serious:** ICD vs physician-activation asymmetry. **D3 Serious:** 27% post-hoc exclusion of screened patients as 'not truly septic' — ITT violation. **D6 Moderate:** Abstract over-calls RR 0.13 without acknowledging methodological limitations.
5	Taj 2022	QI project (pre-post, seasonal) Tanzania N=126	Serious	Moderate	Low	Moderate	Moderate	Low	Serious	Outcome: Mortality (18.7% vs 11.5%, p=0.263) **D1 Serious:** No adjustment despite pervasive baseline imbalances: age 72 vs 50yr (p=0.001), sex (p=0.006), recent healthcare exposure (6.3% vs 35.9%, p<0.001), urosepsis 14.6% vs 1.3%. **D2 Moderate:** SIRS-based prospective ID symmetric. **D4 Moderate:** Outcome data sparse; comorbidities incompletely reported. **D5 Moderate:** Assessors non-blinded in single-centre LMIC setting.
6	Gatewood 2015	Retrospective before-after (QI) USA N=761	Serious	Moderate	Serious	Moderate	Low	Moderate	Serious	Outcome: Mortality (13.3% vs 11.1%, p=0.230) **D1 Serious:** No patient characteristics for mortality cohort; concurrent QI initiatives; secular improvement unaddressed. **D2 Moderate:** ICD vs physician-activation asymmetry — post-intervention pathway captures milder cases. **D3 Serious:** 40% post-intervention case exclusion; 124 retrospectively found to have 'no sepsis identifiable'. **D4 Moderate:** No characteristics reported for mortality cohort. **D6 Moderate:** CMI used as proxy for severity adjustment — not patient-level.
7	Blythe 2022	Population economic analysis (DiD, 48 hospitals) Australia N=2,559	Moderate	Moderate	Moderate	Moderate	Low	Low	Moderate	**Mortality excluded** (too few events, 1.1% baseline). Non-ICU LOS (DiD −20.8h [−36.1, −8.0]) **D1 Moderate:** Aggregate-data only; 47.8% PSP vs 24.2% non-PSP admission increase → case-mix shift toward lower-acuity patients. **D2 Moderate:** Aggregate ICD identification; ICD vs pathway-activation asymmetry. **D3 Moderate:** Hospital capability mismatch (all PSP sites ICU-capable; 24/32 non-PSP without ICU). **D4 Moderate:** Aggregate data — individual patient outcomes cannot be assessed.
8	Pouryahya 2020	Prospective cohort (per-protocol) Australia N=191+17	Serious	Moderate	Serious	Moderate	Low	Moderate	Serious	**Adverse direction** — mortality worse in pathway group (13.5% vs 6.5%); abstract misleads ('no effect on mortality') **D1 Serious:** Pre-intervention group substantially older (78 vs 67yr); no adjustment. **D3 Serious:** only 52/109 post-implementation patients completed pathway; 57 non-completers excluded, selectively enriching protocol group. **D4 Moderate:** 14 patients with unknown mortality data**D6 Moderate:** Abstract does not disclose direction of mortality difference (worse in pathway group).
9	Zia 2023	QI project (PDSA, 3 cycles) Pakistan (LMIC) N=176	Serious	Serious	Serious	Moderate	Low	Low	Serious	**Adverse direction** — mortality worse (10%→11.5% at PDSA-3, p=0.051); abstract describes 'inappreciable variation' without disclosing direction **D1 Serious:** No adjustment; secular improvement and antibiotic stewardship programme run concurrently. **D2 Serious:** Retrospective baseline (ICD-coded) vs prospective clinical activation. **D3/4 Serious:** Very small per-period Ns (~30–50); retrospective vs prospective eligibility mechanisms.
10	McColl 2017	Retrospective cohort (ITS) Australia N=7,754	Moderate	Moderate	Moderate	Low	Low	Low	Moderate	Outcome: In-hospital mortality (aOR 0.52 [0.28–0.97], p=0.04) **D1 Moderate:** 12-covariate MVR (MCMC multiple imputation) — good adjustment but no severity scoring (SOFA/APACHE); secular improvement 2009–2013 partially addressed by ITS design. **D2 Moderate:** Symmetric ICD identification for both periods; ITS approach partially addresses trend. **D3 Moderate:** ICD-coded identification for both periods; baseline audit period interpretation required clarification.

11	**Hayden 2016**	Retrospective before-after USA N=465	Serious	Serious	Serious	Moderate	Low	Low	Serious	**Adverse direction** — mortality worse in pathway group (9.3% pre vs 13.8% post, p=0.38) — extreme ICD vs physician-activation asymmetry **D1 Serious:** No adjustment; prospective pathway identification post-intervention vs ICD-coded retrospective pre-intervention creates fundamentally incomparable cohorts. **D2 Serious:** Pre-period ICD codes identify severe cases only; post-period pathway captures all screen-positive patients. **D3 Serious:** Pathway activation post-intervention includes mild-spectrum cases excluded from pre-intervention ICD cohort. **D4 Moderate:** Lactate data missing from 31% of pre-intervention patients.
12	**Threatt 2020**	DNP QI project (retrospective before-after) USA N=310	Serious	Moderate	Moderate	Moderate	Low	Low	Serious	**Reporting error** — p=0.074 mislabelled as 'significant' in abstract and discussion; mortality result is NOT statistically significant **Outcome:** Mortality (12.1% vs 6.2%, p=0.074); LOS unchanged (p=0.663) **D1 Serious:** No adjustment; no baseline characteristics reported. **D2/3 Moderate:** Symmetric ICD identification; case ascertainment drift (ERNSIT broadening) a moderate concern. **D4 Moderate:** Mortality likely complete from administrative database; no confounder data collected.
13	**Borguezam 2021**	QI project (PDSA, pre-post) Brazil (LMIC) N=1,236	Serious	Moderate	Serious	Moderate	Low	Moderate	Serious	Outcome: In-hospital mortality (59.9% vs 47.6%, p=0.279) **D1 Serious:** No adjustment; equal median SOFA (8.0) both groups is a relative strength but no formal group comparison. **D2 Moderate:** ICD vs physician-activation. **D3 Serious:** Unclear intervention arm grouping; time zero undefined; convenience sampling. **D4 Moderate**: no comorbidity or past medical history data **D6 Moderate:** Abstract incorrectly attributes p<0.001 to mortality; blood culture improvement attributed to SST pathway but driven by bottle restocking.
14	**McDonald 2018**	Retrospective before-after Canada N=148 (ICU subset) [from memory]	Serious	Serious	Serious	Moderate	Low	Low	Serious	Outcome: ICU subgroup only: 40% vs 23.5% (p=0.27, n=42) — full-cohort mortality not reported; full-cohort N=1,010 **D1 Serious:** 3.5-year inter-period gap; no severity adjustment; major changes in sepsis practice during gap **D2 Serious:** ICD vs physician-activation asymmetry. **D3 Serious:** Full-cohort mortality not reported. **D4 Moderate:** ICU subgroup outcome data complete; full-cohort mortality withheld.
15	**Malhotra 2021**	QI project (PDSA, 3-phase) India (LMIC) N=293	Serious	Moderate	Serious	Critical	Moderate	Moderate	Critical	Outcome: 24-hour mortality (31.5% pre vs 7.8% post, p<0.05) — uninterpretable due to 45.4% patient loss **D1 Serious:** No adjustment; WhatsApp-based data collection unreliable. **D2 Moderate**: SST applied to only 51.6% of postintervention patients. **D3 Serious:** Blood culture improvement attributed to SST but due to bottle restocking. **D4 Critical:** 45.4% informative censoring — severe cases transferred to other hospitals. **D5 Moderate:** 24-hour endpoint non-comparable with full in-hospital mortality. **D6 Moderate:** Discussion asserts 'direct correlation' between bundle compliance and mortality.
16	**Noureldeen 2024**	QI project (within-programme before-after) Saudi Arabia N=341	Serious	Serious	Serious	Moderate	Low	Low	Serious	Outcome: 28-day in-hospital mortality (32% vs 21%, p=0.04); SOFA comparable (2.3 vs 2.1, p=0.11) **D1 Serious:** Antibiotic supply chain fix (Q1 2018, 93% of compliance failures) as dominant mechanism — first-period patients systematically undertreated independent of severity; no adjusted comparison. **D2 Serious:** Two periods are not clearly distinguishable strategies; both within-programme phases. **D3 Serious:** First-period patients systematically undertreated due to supply chain failure, not severity. **D4 Moderate**: baseline characteristics not reported by period
17	**Liu 2016**	Retrospective cohort (DiD) 21 hospitals, USA N=18,122	Low	Low	Moderate	Low	Low	Low	Moderate	Outcome: In-hospital mortality (aOR 0.79 [0.65–0.96], p=0.02) All domains Low except D3. Comprehensive multivariable adjustment (LAPS2, COPS2, predicted mortality, 9 covariates + hospital random effect). **D3 Moderate:** ICD-9 discharge coding subject to potential awareness-related drift post-implementation; COPS2 increased across periods (more comorbid patients enrolled post-implementation). [Note: intermediate lactate only (2–4 mmol/L, haemodynamically stable)]
18	**Barbash 2021**	Longitudinal ITS, 11 hospitals USA N=54,225	Low	Moderate	Low	Low	Low	Low	Moderate	Outcome: In-hospital mortality (ITS-adjusted absolute change 0.1 pp [95% CI −0.9 to 1.1], p=0.87). All domains Low except D2. Risk-adjusted ITS (SOFA, Elixhauser, age, infection source, seasonality, hospital fixed effects). **D2 Moderate:** Sepsis-3 definition avoids ICD-9/10 transition bias; but SEP-1-incentivised blood culture ordering increased post-implementation — ascertainment bias documented (expanded denominator, lower average severity post-SEP-1) biasing toward apparent benefit.
19	**Harley 2021**	Prospective cohort + retrospective ICU baseline 12 EDs, Australia N=523+167	Serious	Serious	Serious	Moderate	Low	Low	Serious	**Paediatric**. **Outcome:** ICU subgroup: 7% baseline (12/167) vs 8% prospective (2/24), p=0.999; 14 total ICU mortality events (severely underpowered). 6-month mortality endpoint non-comparable with other studies. **D1–D3 Serious:** ICD-10 coded vs clinical PSP-activated identification; 3.5-year secular gap; no severity scoring. **D4 Moderate:** 6-month mortality complete via Queensland data linkage; severity scores absent for both ICU cohorts.
20	**Moore 2019**	DNP QI project (before-after) USA N=90/91	Serious	Serious	Serious	Serious	Serious	Moderate	Serious	**Mortality not reported. D1 Serious:** No patient characteristics reported; major concurrent confounder. **D2 Serious:** DART checklist drives active screening documentation in intervention period. **D3 Serious:** DART 'code sepsis' announcement directly drives prospective identification. **D4 Serious:** No patient characteristics collected; mortality not reported; no missing data handling. **D5 Serious:** Prospective DART checklist (intervention) vs retrospective EHR chart review (baseline). **D6 Moderate:** mortality is entirely absent from the reported results despite being present in discussion
21	**Papali 2017**	Retrospective before-after, pilot QI Haiti (LMIC) N=166	Serious	Serious	Serious	Moderate	Moderate	Low	Serious	Outcome: In-hospital mortality (24.5% vs 25.8%, p=0.85) **D1 Serious:** No severity adjustment; documented severity imbalances (e.g. encephalopathy). **D2 Serious:** Sepsis recognition rose 1%→34% while sepsis prevalence contracted (9.2%→4.6%). **D3 Serious:** Protocol-improved documentation of GCS and urine output changes who meets severe sepsis eligibility. **D4 Moderate**: Transfer misclassification (~20% each cohort all = survivors) underestimates true mortality; **D5 Moderate:** Asymmetric abstractors between periods (single US intensivist pre vs three Haitian internists post).

22	**Bader 2020**	Pre-post, oncology ED Jordan N=168	Serious	Moderate	Moderate	Moderate	Low	Serious	Serious	**Mortality unreportable** — 11.7% absolute decrease claimed with no raw numerators, no CI, no statistical test; authors explicitly state, 'no analysis was performed to infer causality'. **D1 Serious:** No baseline comparison; illness severity, septic shock, neutropenia, infection source all unmeasured. D2/3 Moderate: Different screening tool and eligibility used pre/post. **D4 Moderate**: mortality outcome data unreported at the individual level **D6 Serious:** Mortality presented as positive finding despite untested, unverified data. Process outcome (triage-to-antibiotic: 95→45 min; 10.8%→89.4% within 1h, p<0.001) is robustly reported.
23	**Mittal 2019**	QI before-after (PDSA) India (LMIC) N=31	Serious	Moderate	Moderate	Moderate	Low	Moderate	Serious	**Paediatric** with mortality numerically worse (11% vs 15%, p=1.0); infant-heavy phase 2 (median age 0.9 vs 5.0yr) explains discrepancy rather than treatment harm. **D1 Serious:** N=31 total; 2 deaths per group — never adequately powered for any clinical outcome; no severity adjustment; divergent infection source distribution (hepatitis 28% vs 0%). **D6 Moderate:** Abstract states 28 and 13 patients were 'included' when these were the numbers excluded. Process outcome (time-to-antibiotic: 50→20 min, p=0.02) is the only meaningful finding.
24	**Bruce 2015**	Retrospective before-after USA N=195	Moderate	Moderate	Moderate	Low	Low	Moderate	Moderate	Outcome: In-hospital mortality (24.2% vs 21.3%, p=0.838) **D1 Moderate:** Statistically significant SBP imbalance at triage (47.5% pre vs 22.4% post hypotension, p=0.003) — pre-protocol cohort substantially more unwell; secular improvement acknowledged by authors and evident in Figure (declining antibiotic times before implementation). **D2/D3 Moderate:** Symmetric ICD-9 identification reduces classic ascertainment asymmetry. **D6 Moderate:** No pre-registration; candid reporting of null result and pre-protocol trend. Null result strengthens review finding of no mortality benefit.
25	**Tromp 2010**	Prospective before-after Netherlands N=825	Moderate	Serious	Serious	Moderate	Low	Moderate	Serious	Outcome: In-hospital mortality (6.3% period 1 vs 5.5% period 3; no p-value reported) **D1 Moderate:** Early SSC rollout era. Broad inclusion (all sepsis; only ~4% septic shock) and low baseline mortality (6.3%) limit comparability. Study not powered for clinical outcomes; no p-value for mortality. **D2/D3 Serious:** Dominant flaw — retrospective discharge-coded identification in Period 1 (n=159) vs prospective nurse screening in Periods 2–3 (n=447); near-tripling of included patients reflects improved case ascertainment, not treatment effect. Declining septic shock prevalence (5.0%→1.8%) confirms progressive enrichment of milder cases. **D6 Moderate:** No p-value for mortality comparison.
26	**Seminari 2023**	Pre-post, BSI-confirmed Italy N=722	Low	Low	Low	Low	Low	Moderate	Moderate	Outcome: In-hospital mortality (18.9% vs 12.7%, p=0.03; adjusted OR 0.64 [95% CI 0.41–0.98]) **D1 Low:** Multivariate logistic regression with backward elimination (age, organism type, neoplasm, diabetes, CKD); SOFA and MEWS comparable (p=0.13, p=0.11). **D2/D3 Low:** Symmetric objective case-finding; no ascertainment asymmetry. **D6 Moderate:** No pre-registration; adjusted and crude results both transparently reported; authors appropriately caution against causal inference. Residual limitation: secular improvement inherent to all pre-post designs.
27	**Song 2019**	Pre-post (i-SMS), tertiary ED South Korea N=631	Low	Low	Low	Low	Low	Low	Low	Outcome: 30-day mortality crude difference (37.3% vs 29.5%, p=0.037) is NULL after adjustment. **D1 Low:** Comprehensive Cox model (SOFA, septic shock, lactate, CRP, age, SSC compliance); identical qSOFA ≥2 trigger in both periods; virtually identical Ns (316 vs 315) confirm equivalent case-finding. **D4 Low:** 30-day outcome complete with active telephone follow-up. **D6 Low:** Exemplary transparent reporting; crude and adjusted results both reported; authors candidly decline causal attribution. Key finding: SSC compliance independently associated with lower mortality (aHR 0.62, p=0.004).
28	**Ruttanaseeha 2020**	Pre-post (SWAT protocol) Thailand N=192	Serious	Serious	Serious	Serious	Moderate	Serious	Serious	Outcome: Mortality (5.3% vs 0.0%, p=0.059); 0 post-protocol deaths in 41.7% septic shock cohort is clinically implausible **D1 Serious:** No adjustment despite profound group non-equivalence: septic shock 41.7% vs 15.6%, qSOFA ≥2: 74% vs 20%, vasopressor use 10.9% vs 3.1%. **D2/D3 Serious:** Dramatic severity distribution shift inconsistent with underlying population; n=192 is ~10% of calculated required sample (n=1,927). **D4 Serious:** Zero deaths in high-severity cohort suggests incomplete mortality ascertainment; follow-up window undefined. **D6 Serious:** Non-significant result (p=0.059) overcalled as benefit in discussion.
29	**Francis 2010**	Retrospective chart review pre-post Canada N=213 (3 teaching EDs)	Moderate	Low	Moderate	Low	Low	Low	Moderate	Mortality not reported — process outcomes only. **Process outcome:** Median time from severe sepsis criteria met to antibiotic: 163→79 min (reduction 84 min, 95% CI 42–126, log-rank p<0.001). **D1 Moderate:** Secular improvement (early SSC era). **D3 Moderate:** Hospital distribution shift (p<0.001) formally tested and excluded by Cox regression. Methodological strengths: blinded abstractors, calibrated extraction, Cox site-confounding test.
30	**Flack 2023**	ITS (antimicrobial stewardship focus) USA N=15,374 all ED patients	Serious	Moderate	Serious	Serious	Low	Low	Serious	No sepsis-specific mortality reported. **D1 Serious:** No comorbidity, severity, or source of infection data; no adjustment; 2-year gap with COVID-19 era confounding. **D2 Moderate:** EMR-based identification of patients by BS antibiotic receipt **D3 Serious:** Post-period BS-antibiotic cohort 47% larger. **D4 Serious:** Critical confounders absent; severity differences. Key narrative finding: sepsis initiative increased BS antibiotic use (3.85%→6.18% of ED visits) and was associated with a doubling of subsequent MDR infection rates (RR 2.0, 95% CI 1.3–3.2, p=0.0009) across the entire ED population.
31	**Venkatesh 2022**	Before-after cohort, 14-hospital Collaborative Australia N=1,802	Moderate	Moderate	Moderate	Low	Low	Low	Moderate	Outcome: In-hospital mortality (11.1% vs 11.7%, aOR 1.1 [95% CI 0.8–1.5]); ICU admission significantly reduced (aOR 0.5 [0.4–0.7]). **D1 Moderate:** Triage category, renal dysfunction imbalance (21.0% vs 13.6%); multivariable regression partially mitigates. **D2/D3 Moderate:** Consistent pathology-system identification; positive blood culture restriction excludes culture-negative sepsis (majority of ED sepsis).

32	**Balamuth 2016**	Concurrent comparison (NOT pre-post) USA N=189	Moderate	Low	Low	Low	Low	Moderate	Moderate	**Paediatric**. **Outcome:** Primary — OD-free by hospital day 2 (aOR 4.23 [95% CI 1.70–10.4], p=0.002). Mortality (3.3% vs 2.9%, p=0.9). **D1 Moderate:** Concurrent same-period design eliminates secular improvement. Residual: unmeasured recognition-complexity confounder (explicitly acknowledged); initial lactate significantly imbalanced (higher in protocol group, p=0.001) and unadjusted; PIM-2 inclusion post-ED therapy introduces over-adjustment. **D6 Moderate:** No pre-registered analysis plan; transparent reporting mitigates.
33	**Narayanan 2016**	Pre-post (SS-BPA electronic alert) USA N=214	Moderate	Moderate	Moderate	Moderate	Moderate	Moderate	Moderate	Outcome: In-hospital mortality (adjusted OR 0.64 [95% CI 0.26–1.57]); LOS significantly reduced (geometric mean ratio 0.66 [95% CI 0.53–0.82]); time to antibiotics 61.5→29 min (p<0.001). **D1 Moderate:** Pre-post without concurrent control; no power calculation; ~2-month wash-in gap is a protective design feature. Education treated as inseparable implementation component. **D2/D3 Moderate:** ICD-9 ascertainment partially mitigated by chart review; non-standard time 0 definition (clinical criteria confirmation, not ED triage) limits comparability. **D4/D5 Moderate:** Raw mortality numerators/denominators not reported. Secular improvement (primary D1 confounder) cannot be excluded, LOS reduction and antibiotic timing improvements more plausibly attributable to intervention. **D6 Moderate:** Absence of registration and absence of raw mortality data for comparison.

Abbreviations: aHR = adjusted hazard ratio; aOR = adjusted odds ratio; BC = blood culture; BSI = bloodstream infection; bpm = beats per minute; CCI = Charlson Comorbidity Index; CI = confidence interval; CKD = chronic kidney disease; COPS2 = Comorbidity Points Score v2; DiD = difference-in-differences; DNP = Doctor of Nursing Practice; ED = emergency department; EHR = electronic health record; GCS = Glasgow Coma Scale; ICU = intensive care unit; ITS = interrupted time series; IV = intravenous; LAPS2 = Laboratory Acute Physiology Score v2; LMIC = low/middle-income country; LOS = length of stay; MAP = mean arterial pressure; MDR = multi-drug resistant; MVR = multivariable regression; OR = odds ratio; PDSA = Plan-Do-Study-Act; PIM-2 = Paediatric Index of Mortality 2; PICU = paediatric intensive care unit; PSP = paediatric sepsis pathway/; RCT = randomised controlled trial; RR = relative risk; SBP = systolic blood pressure; SEP-1 = Medicare Severe Sepsis/Septic Shock Early Management Bundle; SIRS = systemic inflammatory response syndrome; SNAP = SAS Nurse Activated Protocol; SOFA = Sequential Organ Failure Assessment; SS-BPA = Severe Sepsis Best Practice Alert; SSC = Surviving Sepsis Campaign; SST = Sepsis Screening Tool; SWAT = Sepsis Workup and Treatment.
